# Biosynthesis of Bioactive Human Neurotrophic Factor 3 in Silkworms and Its Biomedical Applications

**DOI:** 10.3390/insects16070676

**Published:** 2025-06-27

**Authors:** Wenjing Geng, Liang Lu, Tangmin Li, Mingyi Zhou, Wei Chen, Hao Tan, Debin Zhong, Guanwang Shen, Ping Lin, Qingyou Xia, Ping Zhao, Zhiqing Li

**Affiliations:** 1 Integrative Science Center of Germplasm Creation in Western China (CHONGQING) Science City & Southwest University, Biological Science Research Center, Southwest University, Chongqing 400715, China; 2 Key Laboratory for Germplasm Creation in Upper Reaches of the Yangtze River, Ministry of Agriculture and Rural Affairs, Chongqing 400715, China; 3 Century Legend Biotechnology Research Institute (Chongqing) Co., Ltd., Chongqing 402160, China

**Keywords:** human neurotrophic factor 3, biosynthesis, silk materials, silkworm

## Abstract

Neurotrophic factor 3 (NT-3), a potent neurotrophin, promotes neuronal survival and axonal regeneration while demonstrating a unique capacity to induce lineage-specific differentiation of pluripotent stem cells into functional neurons, underscoring its therapeutic potential in neural repair. Despite these advantages, the large-scale production of recombinant human NT-3 with preserved structure integrity and functional bioactivity remains a critical challenge. This study takes advantage of the silk gland bioreactor of silkworms for the recombinant expression of human NT-3 protein on a large scale. Our findings reveal that NT-3 was successfully expressed in the middle silk gland of silkworms and secreted into the silk fibers, achieving a yield of up to 0.5 mg of bioactive NT-3 per gram of cocoon weight. The engineered NT-3-functionalized silk material demonstrates no cytotoxicity and significantly enhanced the proliferation, migration, and differentiation of neural cells compared to natural silk protein. Importantly, this functionalized material also promotes neurite outgrowth in HT-22 cells. These results collectively underscore the high bioactivity of the recombinant human NT-3 protein produced in the silkworm silk gland. The ongoing fabrication of NT-3-incorporated silk-based materials holds considerable promise for advancing tissue engineering and nerve regeneration applications.

## Simple Summary

The silkworm, *Bombyx mori*, a commercially vital resource for sericulture, produces natural silk with extensive applications spanning textiles, biomedical engineering, and advanced material science. By using the silkworm middle silk gland-specific expression system, we developed a transgenic silkworm strain capable of synthesizing human neurotrophic factor 3 (NT-3) protein, a critical neurotrophic factor essential for neural development, cellular survival, and synaptic plasticity. The resulting NT-3-enriched sericin matrix demonstrated enhanced biological functionality, significantly promoting cell proliferation, migration, differentiation, and neurite outgrowth in mouse hippocampal HT-22 neuron cells. These findings not only establish *B*. *mori* as an efficient bioreactor for large-scale NT-3 production but also reveal a dual function of NT-3 for the preservation of bioactivity and programmable release kinetics, which makes it a promising candidate for treating peripheral neuropathies and spinal cord injuries.

## 1. Introduction

The silkworm, *Bombyx mori*, produces silk fibers through its silk glands, a natural material with broad applications in textiles, medicine, and bioengineering [1]. Known for their ability to produce large quantities of silk proteins and their short growth cycle, silkworms are well-suited for large-scale farming [2]. Silk exhibits exceptional biocompatibility, biodegradability, and mechanical properties, making it an ideal candidate for drug delivery and tissue engineering [3]. The internal environment in the silk gland facilitates proper protein folding and post-translational modifications, thereby enhancing protein activity [4]. Research on silk gland bioreactors focuses on replicating this natural environment to improve silk protein production efficiency and expand its applications into new fields [5,6]. By utilizing a silkworm silk gland bioreactor, silk proteins can be efficiently produced in a controlled setting, significantly reducing the space and time required compared to the traditional *Escherichia coli* expression system [7]. Furthermore, by optimizing environmental factors such as temperature, humidity, and nutrient composition, the synthesis of silk proteins can be fine-tuned to ensure consistent and high-quality output [8].

The application of transgenic technology to modify silkworm silk glands for the production of targeted proteins represents a cutting-edge area of research [9]. By introducing specific genes into silk gland cells, this approach enables silkworms to produce recombinant proteins with greater efficiency and precision [5]. These recombinant proteins can be harvested through the silk-spinning process, significantly improving both the yield and functionality of silk proteins, thereby advancing related industries [10]. The extracted silk proteins containing diverse recombinant proteins have various applications in biomedicine and biomaterials, including sutures, scaffolds, coatings, and drug delivery, exhibiting substantial market potential [11]. Unlike synthetic materials, silkworm silk proteins are naturally derived, highly biodegradable, and more sustainable, aligning with the principles of eco-friendly development [12]. Therefore, research on silk gland bioreactors not only opens new avenues for the silk industry but also highlights their promising potential in biomedical materials and other advanced fields [10,13].

Neurotrophic factors from vertebrates are a group of proteins crucial for the survival, development, and functionality of nerve cells [14,15]. They play a pivotal role in maintaining nervous system health by promoting neuronal growth, differentiation, and repair [14,15]. Among these factors, neurotrophin-3 (NT-3) has obtained significant attention owing to its vital roles in neurodevelopment, neuronal survival, and synaptic plasticity [16]. It supports the growth and survival of diverse neuron types, particularly sensory and motor neurons [17,18]. Previous work has indicated that NT-3 holds promise for treating neurodegenerative diseases, nerve injuries, and facilitating neuroregeneration [19,20]. However, the large-scale production of NT-3 protein remains a significant challenge. The silkworm silk gland bioreactor emerges as a scalable and efficient solution, enabling the mass production of NT-3 protein to meet therapeutic and research demands.

Therefore, in the present work, we developed a transgenic silkworm strain capable of specifically expressing human NT-3 in the middle silk gland (MSG) and efficiently secreting it into cocoon silk. This study established that the recombinant human NT-3 protein was highly expressed in the MSG, achieving a bioactive protein yield of 0.5 mg/g cocoon shell weight. Functional characterization demonstrated that the MSG-derived NT-3 significantly enhanced mouse hippocampal HT-22 neuron viability while promoting cell proliferation, migration, and differentiation. Mechanistic analysis revealed that NT-3-induced neurite outgrowth occurred likely through the activation of GAP43 signaling [21,22]. Taken together, these findings validated the silkworm bioreactor system as an industrially scalable platform for producing bioactive human NT-3 protein, paving the way for the fabrication of NT-3-enriched biomaterials with significant potential for biomedical applications.

## 2. Materials and Methods

### 2.1. Silkworm Strain

The D9L strain silkworms were reared under a photoperiod of 12 h/12 h light/dark at 25 °C utilizing either fresh mulberry leaves or an artificial diet as nutritional substrates. This biological system was subsequently employed for the transgenic expression of recombinant human NT-3 (Accession No. NP_002518) protein.

### 2.2. Cell Line

The mouse hippocampal neuron cell line HT-22 (iCell Bioscience, Shanghai, China) was maintained in Dulbecco’s Modified Eagle’s Medium (DMEM, Gibco, Waltham, MA, USA) supplemented with 10% (*v*/*v*) fetal bovine serum (FBS, Gibco, Waltham, MA, USA). Cell cultures were incubated under standard physiological conditions at 37 °C in a humidified atmosphere containing 5% CO_2_.

### 2.3. Plasmid Construction

The human NT-3 gene sequence was codon-optimized for enhanced expression in silkworm (Table S1). The NT-3 gene, flanked with appropriate restriction sites, was commercially synthesized by BGI (Shenzhen, China). Following synthesis, the NT-3 coding sequence was PCR-amplified using gene-specific primers (Table S2) and subsequently cloned into the *BamH*I/*Not*I-digested sericin-1 promoter sequence cassette of the pSL1180[Hr3Ser1PSer1PA] vector [10]. The resulting recombinant plasmid was then subjected to *Asc*I digestion and subcloned into the piggyBac-based transgenic vector [3 × P3DsRed] to construct the final expression vector pBac[DsRed,NT-3]. All constructs were verified by sequencing.

### 2.4. Transgenic Silkworm Generation

The transgenic construct pBac[DsRed,NT-3] was co-injected with the helper plasmid pHA3PIG at an equimolar ratio (1:1) into the silkworm embryos within 2 h post-oviposition using a microinjection system. The injected G0 embryos were maintained under standard rearing conditions until eclosion, and the resulting adults were intercrossed to generate the G1 progeny. At 6–7 days post-oviposition, G1 embryos were subjected to fluorescence microscopy analysis for initial screening of transgenic individuals based on the 3 × P3 DsRed eye-specific marker expression. Through subsequent rounds of hybridization and phenotypic screening, stable transgenic lines with germline transmission of the NT-3 transgene were established and maintained for further research.

### 2.5. Genetic Analysis

Transgenic silkworm moth genomic DNA was isolated, digested with *Hae*III at 37 °C overnight, and purified using a standard phenol-chloroform extraction protocol. For inverse PCR analysis, 2.5 μg of genomic DNA was circularized by T4 DNA ligase (Takara, Dalian, China) in a total volume of 20 μL at 16 °C overnight. The circularized DNA was then amplified using Taq DNA polymerase (Takara, Dalian, China) with vector-specific primers Reverse-pBac-F and Reverse-pBac-R (Table S2), designed to target the inverted terminal repeat regions of the piggyBac transposon. PCR products were separated on an agarose gel, and target bands were excised and purified using the OMEGA Gel Extraction Kit (Omega Bio-Tek, Guangzhou, China). The purified fragments were cloned into the TA-clone vector (Takara, Dalian, China) for sequencing. Transgene insertion sites were mapped to the silkworm genome using the SilkDB 3.0 database with BLASTN program (https://silkdb.bioinfotoolkits.net/, accessed on 20 December 2023).

### 2.6. Extraction of NT-3 from the Silkworm Cocoon

Recombinant NT-3 protein was extracted from transgenic silkworm cocoons following established protocols with modifications [23,24]. Briefly, cocoon shells were mechanically homogenized and subjected to protein extraction using a denaturing lysis buffer (50 mM Tris-HCl, 8 M urea, pH 7.0) at a solid-to-solution ratio of 30 mg/mL. The extraction was performed at 80 °C for 30 min and the resulting homogenate was clarified by centrifugation at 15,000× *g* for 30 min at 4 °C to remove insoluble debris. For buffer exchange and urea removal, the supernatant was dialyzed against 20 mM phosphate-buffered saline (PBS, pH 7.4) using 3.5 kDa molecular weight cut-off (MWCO) cellulose membranes (Solarbio, Beijing, China) at 4 °C. The dialysis buffer was replaced at 12 h intervals over a 72 h period to ensure complete removal of denaturants while maintaining protein stability.

### 2.7. Quantitative Real-Time PCR Analysis

To quantify the transcriptional expression of the exogenous NT-3 transgene, total RNA was isolated from the MSG of both wild-type (WT) and transgenic silkworms using the E.Z.N.A.^®^ Total RNA Kit (Omega Bio-tek, Guangzhou, China). For cDNA synthesis, 2 μg of total RNA from each sample was reverse-transcribed using the GoScript^TM^ Reverse Transcription System (Promega, Madison, WI, USA). Quantitative real-time PCR (qRT-PCR) analysis was performed in triplicate using the SYBR Premix Ex Taq^TM^ II kit (Takara, Dalian, China) on an Applied Biosystems 7500 Fast Real-Time PCR System (Thermo Fisher, Waltham, MA, USA). The expression of eukaryotic translation initiation factor 4A (eIF-4a) was used as an internal control.

### 2.8. Western Blot Analysis

To assess the expression of recombinant NT-3 protein, protein concentration of the lysate was determined using the Bradford assay with bovine serum albumin (BSA) as the standard. For Western blot analysis, 20 μg of total protein from each sample was resolved by 12% SDS-PAGE under reducing conditions and electrophoretically transferred to a 0.45 μm polyvinylidene difluoride (PVDF) membrane (Millipore, Temecula, CA, USA). The membrane was probed with a rabbit polyclonal anti-NT-3 primary antibody (Abcam, Cambridge, UK; 1:10,000 dilution) overnight at 4 °C, followed by incubation with HRP-conjugated goat anti-rabbit IgG secondary antibody (Beyotime, Shanghai, China; 1:10,000 dilution) for 1 h at room temperature. For the detection of GAP43 expression, HT-22 cells treated with different cocoon extracts were lysed using RIPA cell lysis buffer (Beyotime, Shanghai, China). The extracted proteins were incubated with an anti-GAP43 antibody (Beyotime, Shanghai, China; 1:1000 dilution), and an anti-β-Actin antibody (Beyotime, Shanghai, China; 1:5000 dilution) was used as the internal control. Protein bands were visualized using the ECL Prime Western Blotting Detection Reagent (Amersham Biosciences, Little Chalfont, UK) and imaged using the Chemiscope Series imaging system (Clinx Science Instruments, Shanghai, China).

### 2.9. Quantification of NT-3

To quantitatively assess NT-3 protein expression levels, silver staining analysis was conducted following established protocols with modifications. A series of BSA standards (Beyotime, Shanghai, China) ranging from 100 ng to 800 ng (100 ng, 200 ng, 300 ng, 400 ng, and 800 ng) were co-electrophoresed with 30 μL of experimental protein samples on a 12% SDS-PAGE. Following electrophoresis, proteins were visualized using the Fast Silver Stain Kit (Beyotime, Shanghai, China) according to the manufacturer’s protocol. Quantitative analysis of NT-3 protein bands was performed using ImageJ software (v.1.4.3.67, https://imagej.net/ij/, accessed on 25 June 2025) by establishing a standard curve from the BSA reference bands.

### 2.10. Immunofluorescence Staining

The MSG of silkworms at day 6 of the 5th instar larvae were fixed overnight using 4% (*v*/*v*) paraformaldehyde, embedded in Tissue-Tek^®^ O.C.T.^TM^ compound (Sakura Finetechnical Co., Ltd., Tokyo, Japan), and subsequently sectioned into 7 μm thick slices using a cryostat microtome (Leica, Wetzlar, Germany). The sections were subjected to immunoblotting with a rabbit anti-NT-3 polyclonal antibody (Abcam, Cambridge, UK), followed by detection with CY3-conjugated goat anti-rabbit IgG (Beyotime, Shanghai, China). Fluorescence imaging was captured using a fluorescence microscope (Leica, Wetzlar, Germany).

### 2.11. Scanning Electron Microscopy

The morphological characteristics of both WT and NT-3 silkworm cocoons were analyzed using scanning electron microscopy (SEM). The samples were initially dehydrated in a constant-temperature drying oven, followed by sputter coating with a thin layer of gold (Au) using an MCI000 coater (Tokyo, Japan). The gold-coated specimens were then imaged using a Hitachi SU3500 SEM system (Tokyo, Japan) under optimized operating conditions.

### 2.12. Phenotypic Observation

The cocoon and pupal phenotypes of both WT and NT-3 silkworm strains were observed using a camera (Nikon, Tokyo, Japan). Quantitative analysis of key economic parameters, including total cocoon weight, cocoon shell weight, and pupal weight, was performed for comparative evaluation between the two silkworm strains.

### 2.13. Cell Viability

HT-22 cells were plated in 24-well culture plates at an optimal density and treated with either NT-3 or WT silkworm cocoon extract (100 ng/mL) for 48 h under standard culture conditions. Cell viability was quantitatively evaluated using the Live-Dead^TM^ Viability/Cytotoxicity Assay Kit (Thermo Fisher, Waltham, MA, USA), where metabolically active cells were stained with green-fluorescent Calcein-AM, while non-viable cells were labeled with red-fluorescent propidium iodide (PI). Untreated cells maintained under identical culture conditions served as negative controls. Fluorescence imaging was performed using a laser scanning confocal microscope (ZEISS LSM 880, Oberkochen, Germany) with appropriate filter sets for dual-channel detection.

### 2.14. Cell Proliferation

For EdU staining, HT-22 cells were plated in 24-well culture plates at a density of 1 × 10^4^ cells/well. Following experimental treatments, cells were pulse-labeled with 2 μM BeyoClick^TM^ EdU-488 (Beyotime, Shanghai, China) for 2 h at 37 °C in a humidified 5% CO_2_ atmosphere. Subsequently, cells were fixed and counterstained with 4′,6-diamidino-2-phenylindole (DAPI, Beyotime, Shanghai, China) for nuclear visualization. Fluorescence imaging was performed using a laser scanning confocal microscope (ZEISS LSM 880, Oberkochen, Germany).

For the CCK-8 assay, HT-22 cells were seeded in 96-well microplates at a density of 5 × 10^3^ cells/well and treated with either NT-3 or WT silkworm cocoon extract (100 ng/mL) or NT-3 protein standard (Std) for 24 h under standard culture conditions. Following treatment, 10 μL of Cell Counting Kit-8 (CCK-8; Beyotime, Shanghai, China) reagent was added to each well, and the plates were incubated at 37 °C in a 5% CO_2_ atmosphere for 1 h. The optical density (OD) at 450 nm was measured using a Glomax Multi Detection System (Promega, Madison, WI, USA).

### 2.15. Cell Differentiation

qRT-PCR was performed to evaluate the neurodifferentiation potential of human-derived NT-3 protein-incorporated silk fibroin hydrogel HT-22 cells, which were plated in 24-well culture plates at a density of 1 × 10^4^ cells/well. Following experimental treatments, total RNA was extracted and cDNA was synthetized. The expression profiles of specific neural markers were analyzed, including neuronal markers of neuron-specific enolase (NSE), neuronal class III β-tubulin (TUBB3), and microtubule-associated protein (MAP2); myelination markers of myelin protein zero (MPZ) and myelin basic protein (MBP); and astrocytic marker of glial fibrillary acidic protein (GFAP). Gene expression levels were normalized to GAPDH as an internal control. All experiments were conducted independently with three biological replicates. Primer sequences for all target genes are provided in Table S2.

### 2.16. Wound Healing Assay

For the cell migration assay, HT-22 cells were cultured in 6-well plates until reaching 90–95% confluence. A standardized linear wound was created in the cell monolayer using a 200 μL sterile pipette tip. The culture medium was replaced with low-serum maintenance medium containing 0.25% fetal bovine serum (FBS), and cells were treated with phosphate-buffered saline (PBS) as negative control, NT-3 silkworm cocoon extract (100 ng/mL), WT silkworm cocoon extract (100 ng/mL), or NT-3 Std. Following 24 h of incubation under standard culture conditions, wound closure was monitored using microscopy (Leica, Wetzlar, Germany). Quantitative analysis of cell migration was calculated by the relative wound closure rate using ImageJ software, and three replicates were performed in each treatment.

### 2.17. Neurite Outgrowth Assay

For neurite outgrowth assessment, HT-22 cells following cocoon extract treatments were fixed with 4% paraformaldehyde in PBS for 15 min at room temperature (RT), followed by permeabilization with 0.5% Triton^®^ X-100 in PBS for 20 min at RT. Cells were subsequently stained with Actin-Tracker^TM^ Red-555 (Beyotime, Shanghai, China) for F-actin visualization and DAPI for nuclear staining. Fluorescence images were acquired using a laser scanning confocal microscope (ZEISS LSM 880, Oberkochen, Germany).

### 2.18. Statistical Analysis

Data are presented as the means ± standard deviation (SD) of three independent biological replicates. Statistical significance (*p*-value) was determined using Student’s *t*-test. Statistical significance is denoted as follows: * *p* < 0.05, ** *p* < 0.01, and *** *p* < 0.001.

## 3. Results

### 3.1. Generation of Human NT-3 Expression System in Silkworm

**Figure 1:**
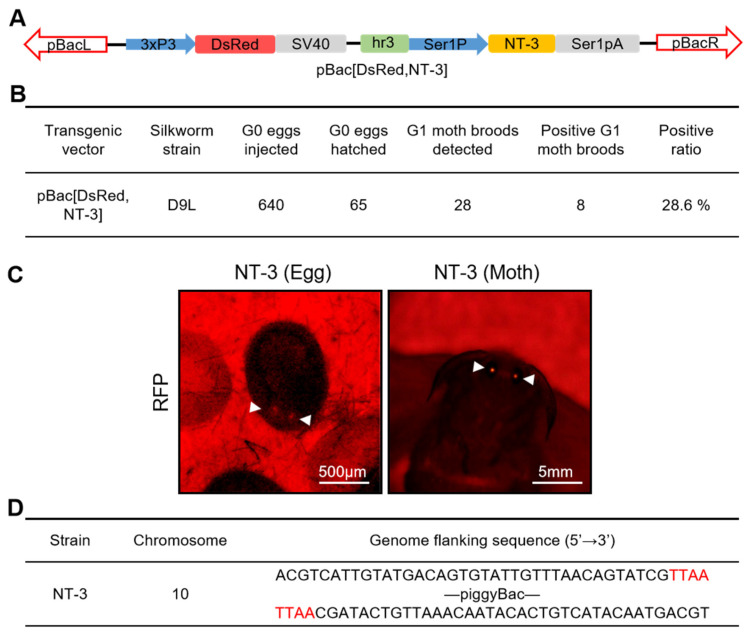
Generation of human NT-3 expression system in silkworm. (**A**) Schematic representation of the pBac[DsRed,NT-3] transgenic expression vector expressed in the MSG of silkworm. (**B**) Germline transformation efficiency of the pBac[DsRed,NT-3] vector upon injection into silkworm embryos. (**C**) Positive G1 eggs and moths were screened under RFP fluorescence (red). The white arrowheads represent the eye fluorescence from embryo and adult. Scale bars are 500 µm and 5 mm, respectively. (**D**) Insertion site analysis of NT-3 gene in transgenic silkworm genome. The target sequence “TTAA”, specifically recognized by piggyBac transposase, was highlighted in red.

To enable the large-scale production of recombinant human NT-3 protein, we established a transgenic expression system using the silkworm silk gland bioreactor. A piggyBac-based transgenic vector for human NT-3 expression was constructed using the MSG expression system [10]. The resulting NT-3 expression plasmid, designated as piggyBac[DsRed,NT-3] (Figure 1A), was co-injected with the transposase helper plasmid pHA3PIG into 640 non-diapause silkworm embryos. Post-injection screening yielded 65 viable larvae that developed into 28 independent G1 broods (Figure 1B). Transgenic candidates were identified through fluorescence screening during key developmental stages: embryos were monitored for ocular fluorescence during body pigmentation, while adults were examined for compound eye fluorescence (Figure 1C). Of these, eight transgenic broods (28.6% transformation efficiency) exhibited stable DsRed marker expression. Inverse PCR analysis confirmed the single-copy integration of the NT-3 expression cassette into chromosome 10 (Figure 1D). All of these data indicate that we have achieved the successful transformation of an NT-3 expression system into the silkworm genome.

### 3.2. Expression of Human NT-3 Protein in Transgenic Silkworm

**Figure 2:**
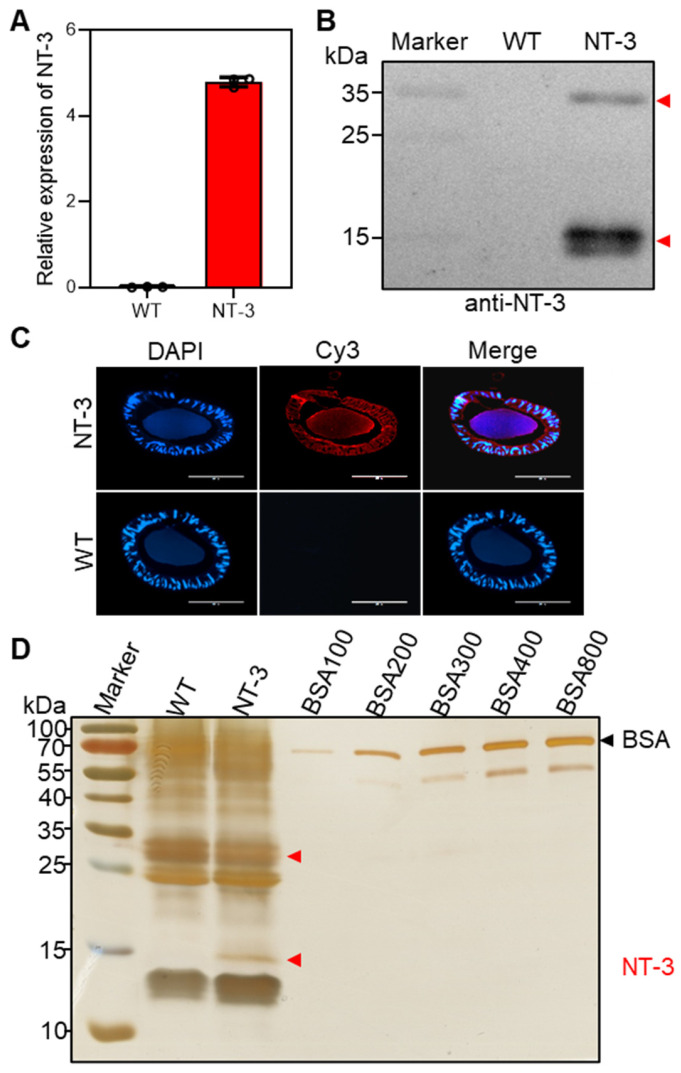
Expression of human NT-3 protein in transgenic silkworm. (**A**) qRT-PCR analysis of NT-3 mRNA transcription in the MSG of silkworm. (**B**) Western blot analysis of NT-3 expression in the MSG using an anti-NT-3 antibody. The red arrowheads represent full-size and active forms of NT-3 protein, respectively. (**C**) Immunofluorescence staining of NT-3 in the MSG using anti-NT-3 antibody (red). Scale bar is 400 µm. (**D**) SDS-PAGE analysis of crude extracts from NT-3 and WT silkworm cocoons to quantify the concentration of NT-3. BSA standard, comprising different concentrations of 100 ng, 200 ng, 300 ng, 400 ng, and 800 ng, was used. The red arrowhead represents active form of NT-3 protein and the black one represents BSA protein.

To characterize NT-3 expression in transgenic silkworms, we performed molecular analyses using the MSG as the target tissue. The total RNA and protein extracts were subjected to qRT-PCR and Western blot analysis, respectively. Both assays demonstrated significant NT-3 expression in the transgenic lines compared to the WT controls (Figure 2A,B). The Western blot analysis revealed two distinct immunoreactive bands at approximately 35 kDa and 15 kDa. The 35 kDa band coincided with the full-length NT-3 protein, while the 15 kDa band represented a proteolytically processed, biologically active form of NT-3 [25]. Immunofluorescence localization studies using anti-NT-3 antibody revealed spatial expression patterns within the MSG. Strong NT-3 signals were observed in the silk gland cells, with evident protein secretion into the sericin layer lumen (Figure 2C). This expression pattern suggested the efficient synthesis and secretion of recombinant NT-3 through the transgenic silk gland expression system.

To quantify the NT-3 protein content in transgenic silkworm cocoons, we extracted the total silk proteins using 1 g of cocoons from at least 20 individual silkworm cocoons as a mixture and analyzed them by SDS-PAGE. The comparative analysis using the Fast Silver Stain Kit revealed a distinct 15 kDa protein band in the transgenic samples, corresponding to the processed NT-3 form, which was absent in the WT controls (Figure 2D). However, the 35 kDa band of the NT-3 protein may not be separated in the NT-3 cocoon extract from the WT extract, presumably attributable to the high abundance of endogenous proteins within the 25–35 kDa molecular weight range. This observation confirmed our previous Western blot results (Figure 2B). For the quantitative analysis, we established a protein standard curve using bovine serum albumin (BSA) at concentrations ranging from 100 ng, 200 ng, 300 ng, 400 ng, and 800 ng. The band intensity quantification and regression analysis enabled the precise determination of the NT-3 content. The transgenic silkworm strain demonstrated substantial NT-3 accumulation, yielding approximately 0.5 mg of bioactive NT-3 per gram of cocoon weight. These findings demonstrate the successful establishment of a stable transgenic silkworm line capable of expressing and secreting human NT-3 protein in the silk gland. The heritable nature of this transgenic system enables continuous propagation, providing a sustainable platform for NT-3 expression.

### 3.3. Effects of NT-3 Expression on Morphological Properties of Silkworm Cocoons

**Figure 3:**
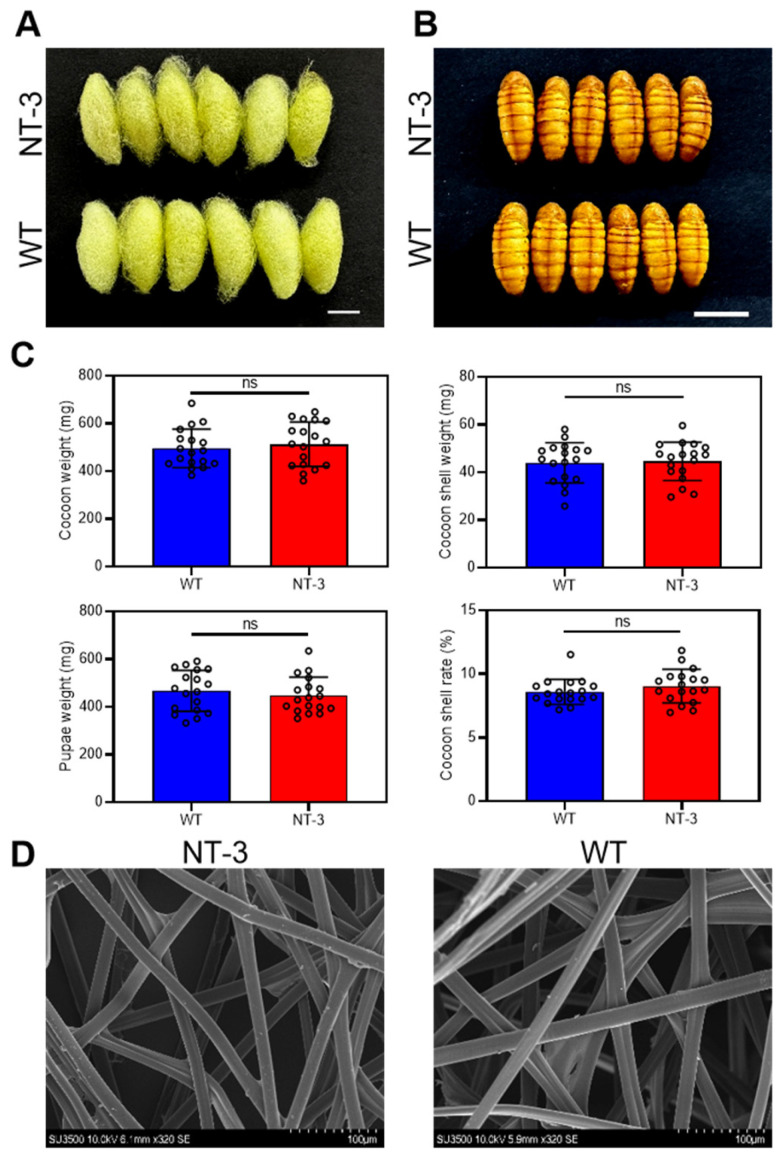
Effects of NT-3 expression on morphological properties of silkworm cocoons. (**A**) Phenotypic comparison of cocoons between WT and NT-3 silkworms. Scale bar is 1 cm. (**B**) Phenotypic comparison of pupae between WT and NT-3 silkworms. Scale bar is 1 cm. (**C**) Impact of NT-3 expression on cocoon weight, cocoon shell weight, pupae weight, and cocoon shell rate. ns: no significance. Data are presented as the means ± SD (n = 18). (**D**) SEM images of silk fibers from WT and NT-3 silkworms. Scale bar is 100 µm.

To assess the potential developmental impacts and economic implications of human NT-3 expression in transgenic silkworms, we conducted comparative analyses of cocoon and pupal characteristics between the WT and NT-3 transgenic lines. The morphological examination revealed no observable differences in the cocoon structure or pupal development (Figure 3A,B). A quantitative evaluation of key economic parameters, including the cocoon weight, shell weight, pupal weight, and cocoon shell rate, demonstrated comparable values between the transgenic and WT lines (Figure 3C). The structural integrity of the transgenic cocoons was further investigated through scanning electron microscopy (SEM) analysis. The NT-3 cocoon shells maintained a native fibroin–sericin architecture, exhibiting microstructure patterns indistinguishable from those of the WT controls (Figure 3D). These comprehensive analyses reveal that human NT-3 expression in the MSG system does not compromise silkworm development or cocoon quality. The preservation of both the developmental parameters and economic traits strongly supports the industrial applicability of this transgenic silkworm system for large-scale NT-3 production.

### 3.4. Cytotoxicity Analysis of Recombinant NT-3 Protein in HT-22 Cells

**Figure 4:**
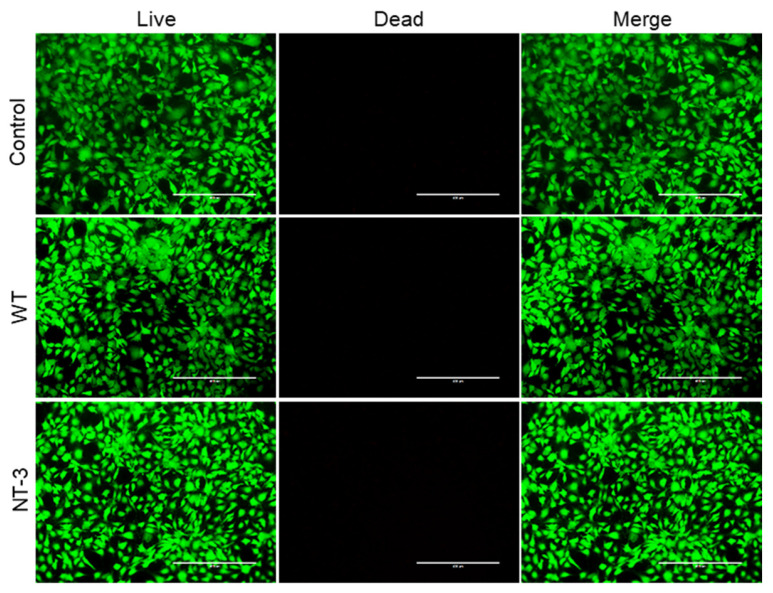
Cytotoxicity analysis of recombinant NT-3 protein in HT-22 cells. Live–Dead cell staining was performed on HT-22 cells treated with cocoon extracts from WT and NT-3 silkworms. Viable cells were labeled with green fluorescence using calcein-AM, while dead cells were stained with red fluorescence using propidium iodide. Scale bar is 400 μm.

Following the successful generation of NT-3-producing transgenic silkworms, we evaluated the biological activity and biocompatibility of the recombinant protein using HT-22 cell cultures. The cell viability assessment through Live–Dead staining revealed comparable cellular survival rates across all experimental groups: untreated controls, WT cocoon hydrogel, and NT-3-enriched cocoon hydrogel (Figure 4). The fluorescence showed minimal dead cell populations in both hydrogel treatment groups, with no significant difference in the viable cell counts compared to the controls. These findings establish two critical properties of the transgenic NT-3 expression system: one is the inherent biocompatibility of silkworm cocoon hydrogel materials, and the other is the absence of cytotoxic effects from the recombinant NT-3 protein. The demonstrated safety profile supports the potential application of this system for further biomedical investigations.

### 3.5. Differentiation Ability of Recombinant NT-3 Protein in HT-22 Cells

**Figure 5:**
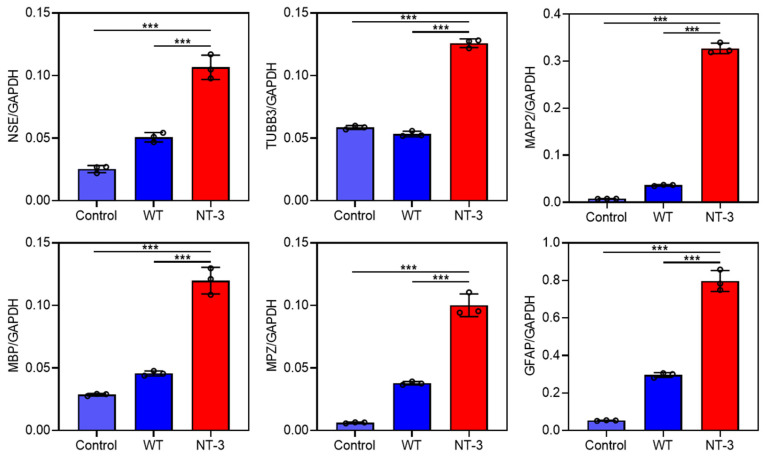
Differentiation ability of recombinant NT-3 protein in HT-22 cells. Cell differentiation assay was performed on HT-22 cells treated with cocoon extracts from WT and NT-3 silkworms. Total RNA was extracted and qRT-PCR was employed to evaluate the expression of neuronal marker genes NSE, TUBB3, and MAP2; myelination markers MPZ and MBP; and astrocyte marker protein GFAP. Data are presented as the means ± SD (n = 3) and analyzed by Student’s *t*-test: *** *p* < 0.001.

To evaluate the biological activity of recombinant NT-3 protein, we investigated its neurodifferentiation potential using gene expression profiling. The qRT-PCR analysis revealed that the treatment with the NT-3-enriched silkworm cocoon extract significantly upregulated multiple neural lineage markers compared to both the untreated controls and WT cocoon extracts (Figure 5). Specifically, we observed the increased expression of neuronal marker genes (NSE, TUBB3, and MAP2), myelin-associated genes (MPZ and MBP), and the astrocytic marker GFAP gene [23]. The above data demonstrate that silkworm-derived recombinant NT-3 protein retains its neurotrophic activity, effectively promoting the expression of neural differentiation marker genes. The observed upregulation of multiple neural lineage markers suggests potential therapeutic applications in neural regeneration and repair.

### 3.6. Proliferation Activity of Recombinant NT-3 Protein in HT-22 Cells

**Figure 6:**
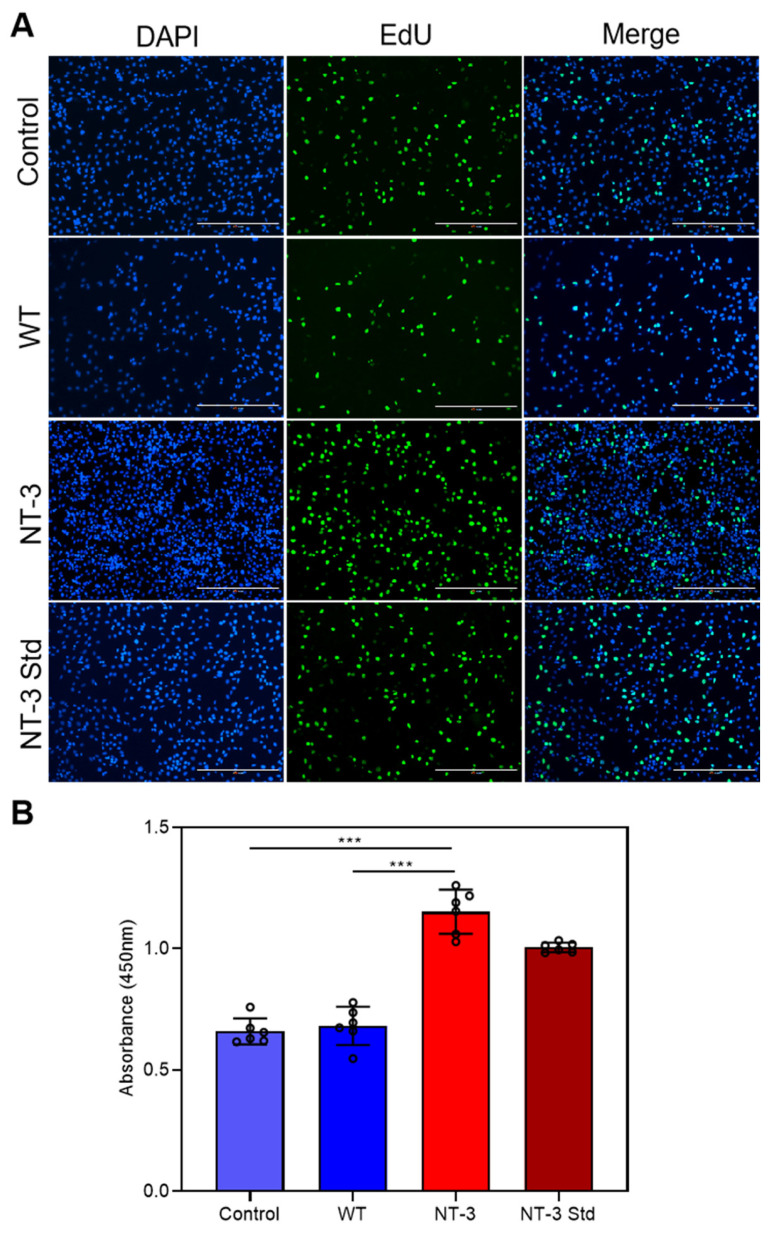
Proliferation activity of recombinant NT-3 protein in HT-22 cells. (**A**) EdU-488 assay was performed to assess the effects of WT and NT-3 cocoon extracts on DNA replication in HT-22 cells after 24 h of treatment (green). Nuclei were counterstained with DAPI (blue). Scale bar is 400 μm. (**B**) Cell proliferation of HT-22 cells treated with WT and NT-3 cocoon extracts for 24 h was evaluated using the CCK-8 assay, with NT-3 Std serving as the positive control. Data are presented as the means ± SD (n = 6) and analyzed by Student’s *t*-test: *** *p* < 0.001.

To further characterize the biological activity of recombinant NT-3, we investigated its mitogenic effects on HT-22 cells using a serum starvation model. The cells were treated for 24 h with either NT-3-enriched cocoon extract, WT cocoon extract, or commercial NT-3 Std. Cell proliferation was assessed through EdU incorporation assays, which detect newly synthesized DNA. Fluorescence microscopy revealed distinct proliferation patterns across the treatment groups. While the control and WT extract-treated cells exhibited minimal EdU incorporation (indicated by faint green fluorescence), both the NT-3 cocoon extract and NT-3 Std treatments exhibited significantly enhanced proliferation, as evidenced by the intense fluorescence signals in a substantially higher number of cells (Figure 6A). Further quantitative analysis using the CCK-8 proliferation assay indicated significant enhancement in cells treated with the NT-3 cocoon extract and commercial NT-3 Std, compared to the WT cocoon extract and untreated controls (Figure 6B). These results provide compelling evidence that the recombinant NT-3 protein produced in the silkworm expression system maintains full biological activity, exhibiting equivalent mitogenic potential to commercially available NT-3 protein in promoting neuronal cell proliferation.

### 3.7. Migration Capacity of Recombinant NT-3 Protein in HT-22 Cells

**Figure 7:**
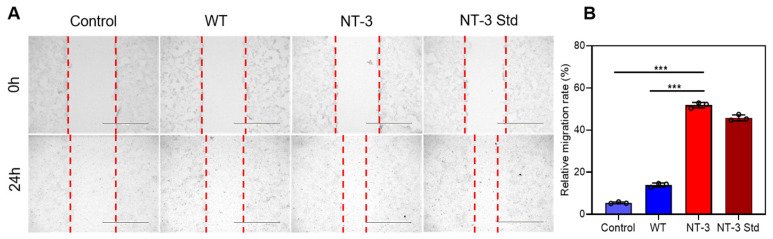
Migration capacity of recombinant NT-3 protein in HT-22 cells. (**A**) A wound healing assay was performed on HT-22 cells treated with cocoon extracts from WT and NT-3 silkworms. Cells were cultured to confluence in six-well plates, and a sterile pipette tip was used to scratch the monolayer. Following the scratch, cells were incubated at 37 °C with the respective treatments for 24 h. Images were captured at 0 and 24 h post-scratch. The red dash lines represent the migration distance of cells. Scale bar is 400 μm. (**B**) The relative migration rate of HT-22 cells under different treatments was quantified. Data are presented as the means ± S.D. with three independent experiments. For the significance analysis, *** *p* < 0.001.

To further elucidate the regulatory effects of recombinant NT-3 on neuronal growth dynamics, the HT-22 cells were incubated for 24 h with either NT-3-enriched silkworm cocoon extract or WT cocoon extract. A wound healing assay was subsequently conducted to evaluate the NT-3-mediated modulation of neural cell migration. Specifically, the scratch widths in the control and WT-treated cells remained markedly larger than those in both the NT-3 cocoon extract and NT-3 Std groups after 24 h, indicating the accelerated migration of NT-3-treated neurons into the space area (Figure 7A). Consistent with this observation, the quantification of migration rates revealed a pronounced enhancement in the NT-3 cocoon extract and NT-3 Std groups relative to the controls (Figure 7B). These findings indicate that recombinant NT-3 potentiates the neuronal migratory capacity, thereby confirming its functional role in promoting neural cell motility.

### 3.8. Neurite Outgrowth of Recombinant NT-3 Protein in HT-22 Cells

**Figure 8:**
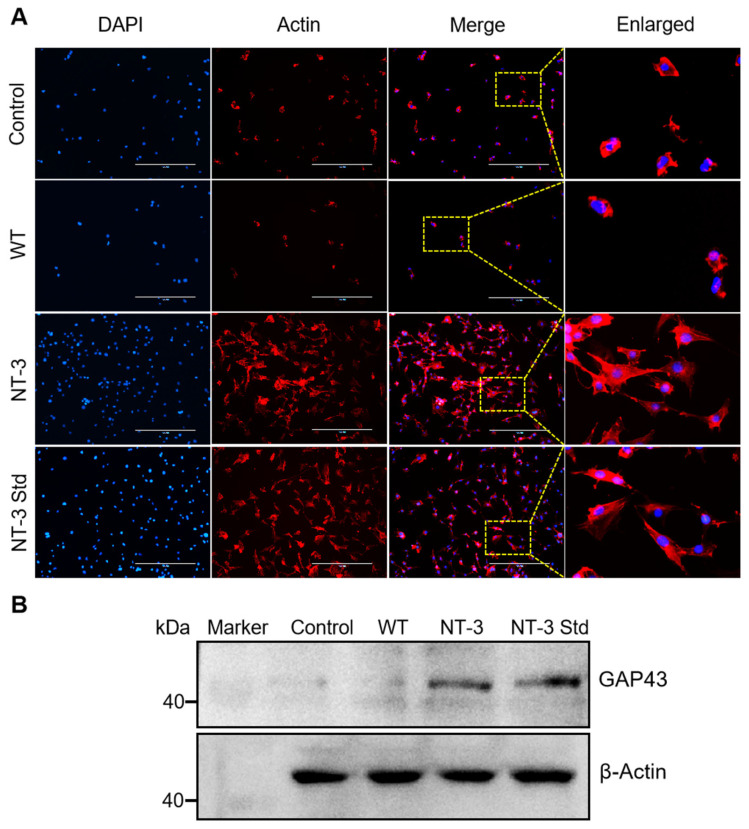
Neurite outgrowth of recombinant NT-3 protein in HT-22 cells. (**A**) HT-22 cells treated with control and NT-3 were immunostained using Actin-Tracker Red to visualize neurites (red), with nuclei counterstained using DAPI (blue). Scale bar is 200 μm. (**B**) The protein expression of GAP43 in HT-22 cells treated with control and NT-3 was analyzed by Western blot. β-Actin served as the internal control.

Beyond its established role in neuronal proliferation and migration, NT-3 was observed to markedly promote neurite outgrowth in the HT-22 cells. To systematically evaluate the contribution of NT-3 to this morphological remodeling, actin cytoskeletal dynamics were analyzed via immunofluorescence staining with Actin-Tracker Red. Fluorescence imaging revealed a substantial increase in neurite complexity and length in the NT-3 cocoon extract and NT-3 Std groups relative to the control and WT (Figure 8A), suggesting NT-3-dependent modulation of cytoskeletal architecture.

Given these morphological alterations, we further investigated the regulatory influence of NT-3 on growth-associated protein 43 (GAP43), a canonical marker of neurite outgrowth implicated in actin cytoskeletal reorganization and axonal elongation [21,22]. A Western blot analysis of the HT-22 cell lysates revealed differential GAP43 expression patterns, with both the recombinant NT-3 and NT-3 std treatments showing significant upregulation compared to that in the untreated controls (Figure 8B). These data collectively establish a mechanistic link between silk gland-derived recombinant NT-3 protein and enhanced neurite outgrowth, mediated through GAP43 signaling activation in HT-22 cells.

## 4. Discussion

NT-3 is a well-characterized neurotrophin factor that plays pivotal roles in neural development, neuronal survival, and synaptic plasticity [17]. Its pleiotropic functions include three primary biological activities: anti-apoptotic regulation to enhance neuronal viability, the facilitation of axonal regeneration following neural injury, and the modulation of synaptic plasticity mechanisms underlying cognitive processes such as learning and memory consolidation [26,27,28]. These multifunctional attributes highlight the therapeutic promise of NT-3 for treating neurodegenerative disorders and facilitating neural repair. Despite this clinical promise, the escalating biomedical demand for bioactive NT-3 and the insufficient yield of functionally active protein from conventional in vitro production systems hinder its applications [29,30]. Consequently, developing innovative bioprocessing strategies for cost-effective, large-scale recombinant NT-3 synthesis represents a crucial unmet need in neurotherapeutic development.

The silkworm has gained prominence as a versatile bioreactor platform due to its specialized silk glands, which enable efficient synthesis and secretion of heterologous proteins into cocoon silk [31]. Moreover, the β-sheet crystalline structure inherent to silk fibers establishes an optimal protective microenvironment for stabilizing exogenous proteins during extended storage. These intrinsic properties endue silk proteins with superior tensile strength, conformational stability, and biocompatibility, which can enhance the structural integrity and functional performance of incorporated recombinant proteins [11,31]. These functional silk protein composites hold significant potential for advanced biomedical applications, including tissue engineering and therapeutic delivery [6,32,33]. Based on this bioengineering framework, we engineered the silkworm silk gland to express recombinant human NT-3 and systematically evaluated the bioactivity of the NT-3-integrated silk proteins.

We successfully generated a transgenic silkworm strain stably expressing NT-3 under the control of the MSG-specific promoter. The quantitative analysis revealed that NT-3 could be secreted into cocoon silk at a yield of 0.5 mg of bioactive NT-3 per gram of cocoon weight, which was comparable with the *E*. *coli* expression system that produced 1.5–3.0 mg human NT-3 protein in 1 mL culture medium [34]. Notably, constitutive NT-3 expression exhibited no observable developmental toxicity to silkworm. Moreover, transgenic silk cocoons retained their native structural integrity, with no significant differences in the cocoon weight or silk microstructure compared to those of the WT silkworm strain. These results collectively demonstrate the feasibility of silkworm as a scalable, cost-effective bioproduction platform for recombinant NT-3 synthesis without compromising silk quality or insect viability.

To validate the bioactivity of silkworm-derived recombinant human NT-3, we performed comprehensive functional assays using HT-22 hippocampal neuronal cells. The EdU incorporation assays demonstrated that our NT-3 significantly increased cell proliferation, with an efficacy comparable to a commercial NT-3 standard. Furthermore, the scratch-wound healing assay revealed great improvements in the neuronal migration velocity with the NT-3 treatment. These functional outcomes, including proliferation potentiation, motility enhancement, and differentiation induction, align mechanistically with the reported roles of NT-3 in synaptogenesis and neural circuit maturation [35,36]. Importantly, the functional equivalence between silkworm-produced NT-3 and commercial standard confirms the preservation of structural and functional fidelity in our silkworm expression system.

Previous studies have demonstrated that NT-3 is upregulated following nerve injury to facilitate nerve regeneration [37,38]. Given the crucial role of neurogenesis in neuronal repair and synaptic plasticity [39], we evaluated the neurotrophic effects of recombinant NT-3 on this process. Our quantitative analysis demonstrated that the HT-22 neuronal cells treated with recombinant NT-3 showed obvious increases in neurite length compared to both the untreated controls and WT protein groups. The Western blot analysis confirmed this functional response, revealing a concomitant upregulation of GAP43, a well-established molecular marker of axonal growth [22]. These findings indicate that NT-3 promotes neurite outgrowth through GAP43-dependent signaling pathways [40], although the precise downstream mechanisms require further investigation.

Our silkworm-based expression system enables the scalable production of bioactive NT-3 while maintaining inherent processability for diverse biomedical applications. The resulting recombinant silk can be fabricated into various formats including hydrogels, films, porous sponges, and 3D scaffolds. A key translational challenge involves overcoming anatomical barriers, such as the blood–nerve interface, to achieve targeted NT-3 delivery at nerve injury sites. To address this, we propose engineering silk biomaterials, particularly enzymatically degradable hydrogels with tunable porosity, to enable spatiotemporally controlled NT-3 release. Future work will focus on the fabrication of different NT-3-containing silk materials and the validation of NT-3 bioactivity in preclinical models of neurodegeneration. By integrating the structural versatility of silk with the neural regenerative property of NT-3, this approach may advance therapies for conditions requiring precise neurotrophic support, such as peripheral neuropathies and spinal cord injuries.

## 5. Conclusions

In this study, we have established a transgenic silkworm platform for the high-yield production of bioactive recombinant human NT-3 protein through an MSG-specific expression system. The NT-3 containing silk materials were shown to enhance cell proliferation, migration, and differentiation, and promote neurite outgrowth by activating GAP43 signaling. These silk-derived NT-3 materials offer pleiotropic functions for bioactivity and programmable release kinetics, which hold considerable promise for advancing tissue engineering and nerve regeneration applications.

## Data Availability

The original contributions presented in the study are included in the article, further inquiries can be directed to the corresponding author.

## Supplementary Materials

The following supporting information can be downloaded at https://www.mdpi.com/article/10.3390/insects16070676/s1: Table S1: The optimized coding sequence of human NT-3 gene according to the silkworm codon usage bias; Table S2: List of primers used in this study.
