# Supplementary material for: Biosynthesis of Bioactive Human Neurotrophic Factor 3 in Silkworms and Its Biomedical Applications
Source: Insects. 2025 Jun 27;16(7):676. doi: 10.3390/insects16070676 (PMC12295520; doi:10.3390/insects16070676)
Supplement: Supplementary file 1 [file insects-16-00676-s001.zip › insects-3620685-supplementary.pdf]

Table S1. The optimized coding sequence of human NT-3 gene according to the silkworm codon usage bias.

Gene name	Sequence (5'-3')
NT-3	GGATCCATGAGCATCCTGTTCTACGTGATCTTCCTGGCTTACC<br>TGAGAGGTATCCAAGGTAACAACATGGACCAAAGATCATTG<br>CCTGAAGACTCACTGAACTCACTGATCATCAAACTGATCCAA<br>GCTGACATCCTGAAAAACAAGCTGTCAAAGCAAATGGTGGA<br>CGTGAAAGAAAACTACCAAAGCACTCTGCCTAAAGCTGAAG<br>CTCCTAGAGAACCTGAAAGAGGTGGTCCTGCTAAATCAGCT<br>TTCCAACCTGTGATCGCTATGGACACAGAACTGCTGAGACA<br>ACAAAGAAGATACAACTCACCTAGAGTGCTGCTGTCAGACT<br>CAACACCTCTGGAACCTCCTCCTCTGTACCTGATGGAAGACT<br>ACGTGGGTTCACCTGTGGTGGCTAACAGAACATCACGCAGA<br>AAGAGATACGCCGAACACAAATCACACAGAGGTGAATACTC<br>AGTGTGCGACTCAGAATCACTGTGGGTGACAGACAAATCAT<br>CAGCTATCGACATCAGAGGTCACCAAGTGACAGTGCTGGGT<br>GAAATCAAAACAGGTAACTCACCTGTGAAACAGTACTTCTA<br>CGAAACACGCTGCAAAGAAGCTAGACCTGTGAAAAACGGT<br>TGCAGAGGTATCGACGACAAACACTGGAACTCACAGTGCAA<br>AACATCACAGACATACGTGAGAGCTCTGACATCAGAAAACA<br>ACAAACTGGTGGGTTGGAGATGGATCAGAATCGACACATCA<br>TGCGTGTGCGCTCTGTCAAGAAAAATCGGTAGAA**CACACCA**<br>**CCACCACCACCACCACCAC**TAAGCGGCCGC

Underlines represent the BamHI/NotI restriction endonuclease sites; Bold letters represent the His-tag sequence.

Table S2. List of primers used in this study.

Gene name	Sequence (5'-3')
NT-3-ORF-F	GGATCCATGAGCATCCTGTTCTACGTG
NT-3-ORF-R	GCGGCCGCTTAGTGGTGGTGGTGGTGGT
Reverse-pBac-F	TACGCATGATTATCTTTAACGTA
Reverse-pBac-R	GTACTGTCATCTGATGTACCAGG
NT-3-qPCR-F	CGCCGAACACAAATCACACA
NT-3-qPCR-R	TTCACCCAGCACTGTCACTT
eIF-4a-qPCR-F	TTCGTACTGGCTCTTCTCGT
eIF-4a-qPCR-R	CAAAGTTGATAGCAATTCCCT
NSE-qPCR-F	GGTCCAAGTTCACAGCCAAT
NSE-qPCR-R	ATGAACGTGTCCTCGGTTTC
TUBB3-qPCR-F	TCCGAGTACCAGCAGTACCA
TUBB3-qPCR-R	TCACTTGGGGCCCTGGGCCT
MAP2-qPCR-F	ACCAACCACTGCCAGACCT
MAP2-qPCR-R	GTGGCGGATGTTCTTCAGAG
MPZ-qPCR-F	ATCGATGAGGTGGGGACCTT
MPZ-qPCR-R	CCACTATGTCCGGTGGGTTT
MBP-qPCR-F	CATCCTTGACTCCATCGGGC
MBP-qPCR-R	TTGTACATGTTGCACAGCCC
GFAP-qPCR-F	CACGAACGAGTCCCTAGAGC
GFAP-qPCR-R	ATGGTGATGCGGTTTTCTTC
GAPDH-qPCR-F	GGACCTGACCTGCCGTCTAG
GAPDH-qPCR-R	GTAGCCCAGGATGCCCTTGA
